# Rethinking Leptospirosis Prevention, the Philippines

**DOI:** 10.3201/eid3203.251250

**Published:** 2026-03

**Authors:** Ryan V. Labana

**Affiliations:** Polytechnic University of the Philippines, Manila, the Philippines

**Keywords:** leptospirosis, Leptospira interrogans, bacteria, bacterial infections, zoonoses, One Health, health governance, Philippines

## Abstract

Leptospirosis, the disease caused by infection with *Leptospira* spp. bacteria, remains a recurring public health challenge in the Philippines, particularly during monsoon floods and typhoon seasons. Despite responsive measures, such as Code White Alerts, standardized treatment protocols, and postflood prophylaxis, cases and associated deaths persist, emphasizing the limitations of reactive strategies. Structural challenges in flood control, urban sanitation, and rodent management hinder long-term prevention. This policy review applies a systems thinking approach to integrate national programs with community-led interventions, recognizing the interlinked roles of environmental management, behavioral change, and grassroots surveillance. Low-cost, context-sensitive actions, such as community drainage clearing, shared protective gear, local rodent-proofing, and barangay-level reporting, can address immediate risks while reinforcing structural initiatives. Embedding those actions within a feedback loop between local actions and national policies fosters resilience, reduces disease incidence, and shifts the paradigm from reactive response to sustainable prevention.

The Philippines usually encounters leptospirosis outbreaks weeks after monsoon floods. In 2025, the Department of Health (DOH) recorded 3,037 cases during January–July; 1,114 of those cases occurred 1 week after the rainy season officially began on June 2. During June 8–August 7, a total of 2,396 verified hospital reports were recorded nationwide. Metro Manila bore the brunt of the impact, and several tertiary hospitals reported strain on capacity caused by the sudden increase in leptospirosis cases. The looming threat of further rainfall could lead to new infections, highlighting the urgent need for early consultation for fever, myalgia, jaundice, and other symptoms after exposure to floodwater (*1*–*4*).

The spikes in leptospirosis cases during typhoon seasons in the Philippines have already been observed in the past several years. For example, Tropical Storm Washi (locally known as Sendong) in December 2011 triggered a poststorm outbreak of leptospirosis. That outbreak resulted in >400 infections and 22 deaths shortly after the floods subsided (*5*). In September 2009, Typhoon Ondoy (Ketsana) caused widespread flooding across Metro Manila, leading to 2,089 leptospirosis cases and 162 deaths; although that event predates 2012, it established a troubling precedent for disease risk tied to typhoons (*6*). 

Epidemiologic evidence shows that leptospirosis hospital admissions typically peak ≈2 weeks after periods of intense rain. However, once the variable of flooding is factored in, the direct rain–leptospirosis association weakens, highlighting flooding itself as the critical driver of transmission (*7*). Flooding increases human contact with water and soil contaminated by the urine of infected rats, which serve as the main reservoirs of *Leptospira* spp. bacteria, the causative agent of leptospirosis (*4*). Case–control studies confirm that contact with floodwater is a significant risk factor; 1 meta-analysis indicated an odds ratio of 2.19 (95% CI 1.48–3.24) for leptospirosis among persons exposed to flooding (*8*). In urban settings, particularly in densely populated slum areas with poor sanitation infrastructure, flood events drastically increase exposure risk for leptospirosis outbreaks (*9*). The consistent pattern across multiple years underscores how every typhoon season, from 2012 on, is a dangerous period for potential leptospirosis outbreaks, particularly in flood-prone urban areas where exposure to *Leptospira*-contaminated water is frequent.

The goal of this policy review is to reframe leptospirosis prevention in the Philippines. By using a systems thinking approach, we examined current national and local interventions to identify strategic leverage points for improvement. Our findings highlight the importance of integrating community-led actions into existing national programs to strengthen feasibility and impact. We advocate for a participatory governance approach that links grassroots engagement with national and local policy, infrastructure, and research toward a more adaptive and resilient framework for leptospirosis control.

## When Interventions Fall Short—Flood Control Controversies, Misguided Education Campaigns, and the Struggle against Leptospirosis

**Figure 1:**
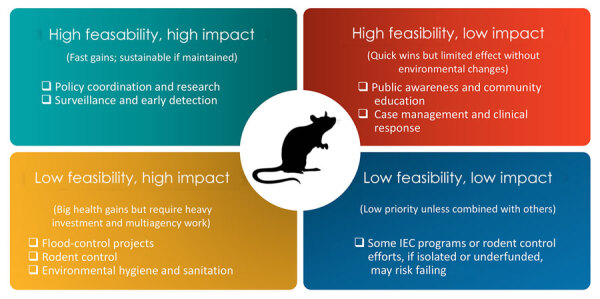
Impact–feasibility matrix of leptospirosis interventions, the Philippines. IEC, information, education, and communication.

Various interventions against leptospirosis exist in the Philippines. They are mapped in this policy review according to feasibility and potential impact, highlighting where existing strategies stand and where improvements are most needed. We summarize the impact–feasibility matrix of leptospirosis interventions in the Philippines (Figure 1).

### High Impact, High Feasibility Interventions

Health policy coordination, especially during a disease outbreak, is a crucial measure to ensure that leptospirosis cases are addressed promptly. The government implements Code White Alerts during peak risk periods. This alert system is used by DOH to enhance the readiness of hospitals and health facilities while they remain on standby for potential case surges. The announcement of a Code White Alert is communicated through press releases and regional and local rollouts. DOH, through its Regional Epidemiology and Surveillance Units, actively tracks and reports cases, whereas local government units collaborate with DOH on real-time data reporting and flood-season mapping to guide targeted interventions. Those programs are crucial in reducing disease severity, preventing fatalities, and ensuring timely clinical care for diagnosed patients, helping them avoid further complications (*10*,*11*). The interventions have high feasibility and high impact in the Philippines.

### High Feasibility, Low Impact Interventions

In the Philippines, the onset of typhoons and monsoon floods often triggers a surge in information, education, and communication (IEC) materials on leptospirosis from government agencies, local government units, universities, and nongovernment organizations; one such IEC material, posted on social media, informed the public about the transmission cycle of *Leptospira* and preventive and control measures (Appendix). That IEC material and other IEC materials, typically focus on ideal preventive actions such as avoiding wading in floodwater, wearing protective boots and gloves, washing with soap after exposure, and seeking early medical care if symptomatic. Although such advisories are medically sound, they often fail to account for the lived realities of urban flood-prone communities. In low-income settlements, many residents have no choice but to wade through floodwaters to evacuate, retrieve belongings, commute to work, or access basic needs. High flood levels make the use of boots impractical. Similarly, hygiene recommendations such as thorough washing with soap and clean water are challenging when water supplies are disrupted and soap is a low priority in emergencies. Messages about rodent control and drainage maintenance may also be irrelevant in the immediate aftermath of a disaster, when survival and shelter take precedence over environmental interventions. The mismatch between the prescriptive tone of IEC materials and vulnerable communities’ structural constraints may reduce the credibility and impact of public health messaging. To be effective, leptospirosis prevention campaigns must move beyond one-size-fits-all advice and integrate context-sensitive strategies, such as guidance for unavoidable exposure, accessible postexposure prophylaxis, and community-based flood safety measures, that reflect the realities on the ground.

For case management and clinical response, standardized Clinical Practice Guidelines developed by DOH with professional medical societies ensure uniform diagnosis and treatment protocols across health facilities (*12*). Benefit packages for leptospirosis, implemented by the Philippine Health Insurance Corporation (PhilHealth), help ease the financial burden on affected patients and support the cost of care by covering expenses related to hospital confinement, laboratory tests, and medications (*13*). The government also deploys medical missions and provides postflood doxycycline prophylaxis to high-risk persons in flood-affected areas. However, when those measures are activated, persons have already been exposed and infected, meaning the burden on the healthcare system and communities remains high.

Educational campaigns with structural constraints do not substantially help in reducing leptospirosis prevalence. On the other hand, case management and clinical response minimize fatalities and severe cases, but do not necessarily ease the burden on the healthcare system. Thus, both are feasible in the Philippines, but both have a low impact.

### Low Feasibility, High Impact Interventions

Flooding, as a major risk factor of leptospirosis outbreaks, requires mitigation, including national flood-control projects. Flood control in the Philippines, particularly in urban communities like Metro Manila, faces chronic challenges from rapid, unplanned urbanization, inadequate infrastructure, and governance issues. Informal settlements along rivers and drainage systems obstruct waterways, whereas aging pumping stations and silted floodways like the Manggahan Floodway in Pasig City struggle to cope with intense rainfall and typhoon-induced surges.

Major projects intended to address these problems often have been marred by controversy. In a city in the Visayas region, for example, nearly 4 billion Philippine pesos’ worth of flood-control projects were reported in 2025 as incomplete, poorly built, or entirely missing. National audits have long indicated that many flood-control budgets are lost to graft, leading to stalled or substandard infrastructure (*14*,*15*). The head of government raised allegations of corruption and mismanagement from the executive and legislative branches, prompting nationwide investigations into several of those projects (*16*).

In a different aspect, Laguna Lakeshore Expressway–Dike, another flood-control project, has faced opposition from some environmentalists and fisherfolk because of concerns about displacement, ecologic damage, and inequitable benefits (*17*). Similarly, the proposed Pasig River Expressway drew criticism for threatening heritage areas, worsening urban heat, and potentially increasing flood risk by reducing river buffer zones (*18*). Even long-standing flood-control structures like the Manggahan Floodway have had unintended consequences, such as aggravating flooding in downstream communities around Laguna de Bay because of siltation and pollution (*19*). Such controversies hamper engineering capacity, transparent governance, inclusive urban planning, and sustained maintenance, making the affected areas vulnerable to recurring, often catastrophic floods.

Although the flood-control projects in the Philippines are not yet adequately managed, other interventions also must be considered. Rat-catching campaigns in the Philippines have long been promoted to reduce leptospirosis and other rodentborne zoonotic diseases, particularly in flood-prone urban and agricultural areas where human–rat contact is frequent (*20*,*21*). However, their long-term effectiveness is challenged by the biology of rats, which have exceptionally high reproductive potential. Many common rat species, such as *Rattus norvegicus* and *Rattus rattus*, reach sexual maturity in as little as 3 months, and female rats can conceive again within weeks after giving birth. Because each litter may produce 6–12 offspring and multiple litters can occur annually, rat populations can rebound quickly even after intensive control efforts (*22*,*23*). This rapid turnover means that without sustained, integrated control, combining environmental sanitation, secure food storage, habitat destruction, and public health education, rat-catching alone offers only temporary relief. The importance of sustained, integrated control cannot be overstated, given that it is the key to long-term prevention and control of leptospirosis transmission, particularly after flooding events when contaminated water is widespread.

Environmental hygiene, an approach that can be integrated with rat-catching, also faces substantial challenges in urban communities, especially in low-income areas of the Philippines. Many urban low-income families live in informal settlements where water access is limited to shared or purchased sources, often of questionable quality (*24*). Housing frequently does not meet the family’s needs, being makeshift, overcrowded, and vulnerable to flooding, creating ideal habitats for rats and other disease vectors (*25*). Waste-management systems are inconsistent or absent, accumulating garbage in streets and waterways, attracting rodents, and exacerbating flooding. Toilet facilities, if present, often are shared by multiple households and may lack proper sewage connections, resulting in environmental contamination that increases the risk for leptospirosis and other zoonotic diseases (*26*). Those constraints mean that, even when communities engage in rat-catching activities, unhygienic environmental conditions continually undermine disease-control efforts, making sustainable prevention difficult without broader improvements in urban infrastructure and social services.

Flood control, rodent control, and environmental hygiene and sanitation are key interventions for mitigating leptospirosis cases and preventing outbreaks. Their collective impact is considerable because they directly disrupt the transmission cycle of *Leptospira* bacteria. However, because of poverty, corruption, and a lack of political will, the feasibility of these essential interventions remains low in the country.

### Low Feasibility, Low Impact Interventions

Some interventions are challenging to implement and yield minimal benefits when executed. For example, developing targeted educational materials for leptospirosis prevention can be challenging because of the diverse and complex social structures across Philippine communities. Cultural variations, language barriers, and differing levels of health literacy make designing messages that resonate effectively with all audiences difficult. In addition, when targeted educational materials are used in isolation, they cannot produce a high impact on reducing leptospirosis transmission. This limitation also applies to rodent control, which will remain ineffective without the integration of improved environmental hygiene and sanitation.

The limited effectiveness of isolated, reactive interventions highlights the importance of prioritizing preventive, integrated strategies over responses. Preventive measures, such as effective flood control, rodent population management, improved urban sanitation, access to protective equipment, and health education before flood events, address the root causes of leptospirosis transmission. Unlike responsive measures, which are reactive and resource-intensive, preventive strategies can reduce exposure risk, lower the incidence of infection, and minimize the need for costly hospitalization and emergency care. In the long term, prioritizing prevention saves lives, alleviates pressure on the healthcare system, conserves public funds, and fosters healthier, more resilient communities.

## Rethinking Leptospirosis Prevention through Systems Thinking

**Figure 2:**
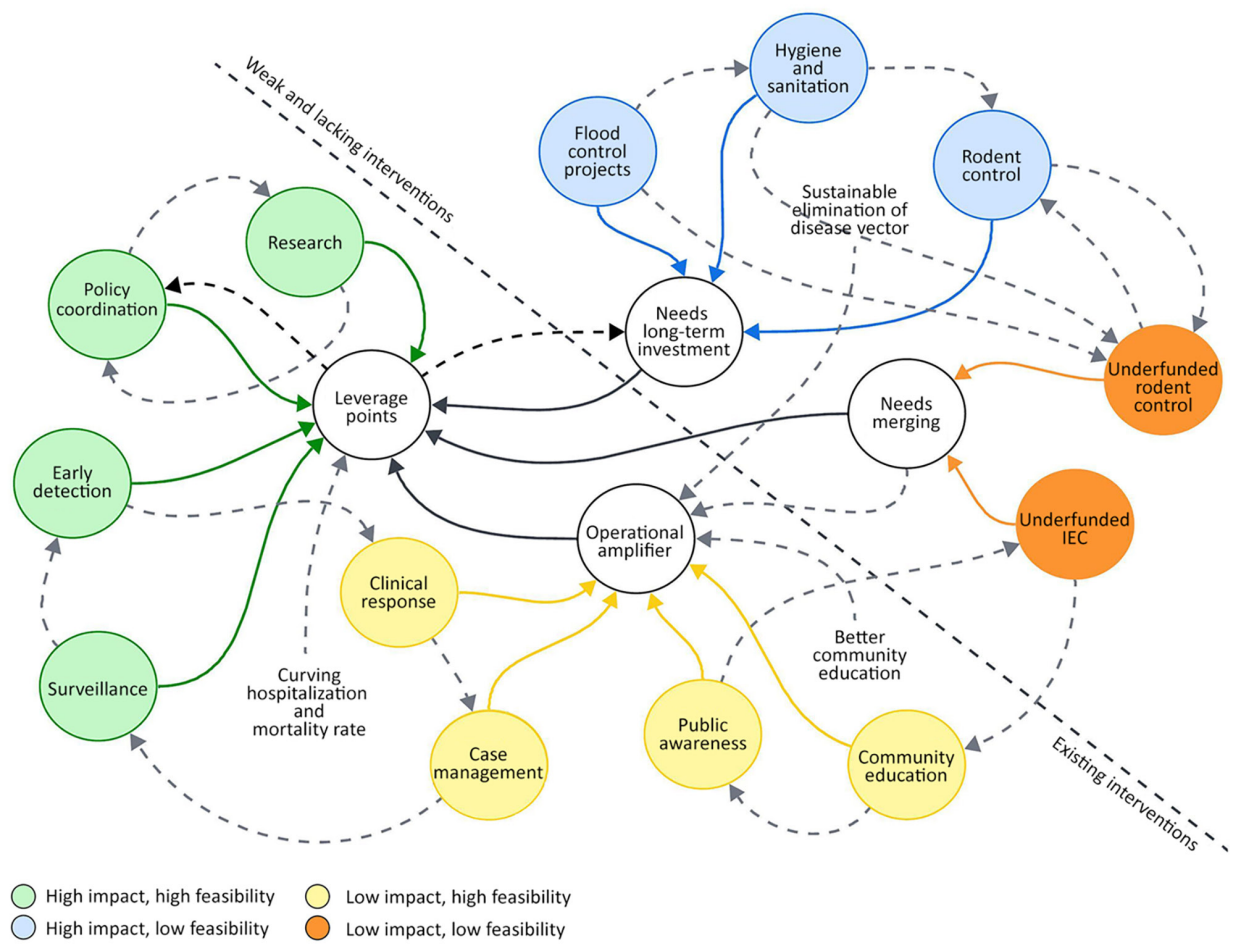
Systems thinking framework linking national strategies for leptospirosis prevention, the Philippines. IEC, information, education, and communication.

National and regional interventions against leptospirosis in the Philippines are often costly, long-term, and dependent on government agencies and multisectoral collaboration. Through use of the impact-feasibility matrix discussed in this policy review, various interventions should be strategically deployed to achieve better outcomes. We used a systems thinking approach, mapping various interventions against leptospirosis to guide and support decision-making for curbing leptospirosis cases in the Philippines (Figure 2).

The systems thinking map (Figure 2) provides a holistic view of how different interventions are interconnected to improve leptospirosis prevention outcomes. At the core of the system are leverage points. They represent critical areas of interventions that have both high impact and high feasibility in the Philippine context. Those areas include policy coordination, surveillance, early detection, and research, all of which strengthen the foundation of leptospirosis management in the country. Surrounding those areas are interventions with moderate impact, such as clinical response, case management, public awareness, and community education, which act as an operational amplifier. That operational amplifier enhances the system’s ability to reduce disease transmission and severity through informed communities and effective healthcare delivery. Meanwhile, flood-control projects, hygiene and sanitation, and rodent control have high impact but are less feasible measures that demand long-term investment and intersectoral collaboration to sustainably reduce environmental risks. Those interventions must be integrated as part of the leverage points for better outcomes. Furthermore, fragmented IEC and underfunded rodent control, which both exist in the Philippines, are considered weak and low-impact components. They require merging and capacity strengthening to become more effective. The map (Figure 2) also illustrates that a successful leptospirosis-prevention strategy depends on reinforcing these interconnected elements.

## Community-Led Approach in Mitigating Leptospirosis Cases in Small Communities

**Figure 3:**
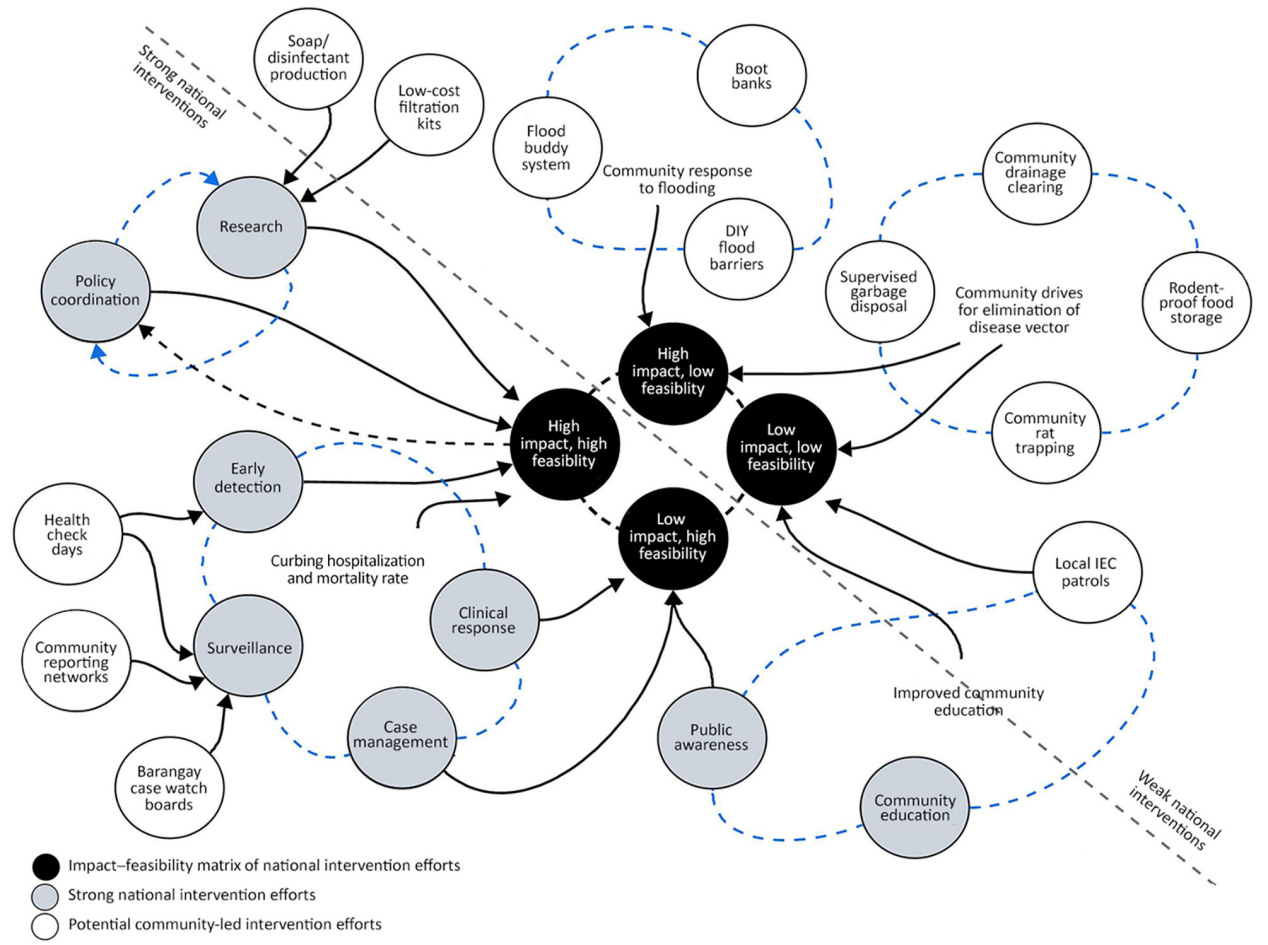
Reframing leptospirosis interventions from national scope into community-led interventions, the Philippines. IEC, information, education, and communication.

Addressing leptospirosis in the Philippines remains a big challenge because of its massive cost in controlling its main drivers: flooding, poor environmental hygiene, and poor sanitation. As a quick-fix solution, with potential to become a sustainable response, community-led interventions can be implemented. Integrating community-led interventions against leptospirosis offers a practical, low-cost, and context-sensitive approach, and it complements national and regional strategies (Figure 3). Such initiatives empower residents to act as the first line of defense, tackling risk factors directly in their environments. Environmental and floodwater management measures, such as monthly drainage-clearing days, DIY flood barriers using sandbags or bamboo, and household-level water diversion, reduce floodwater stagnation and limit contact with contaminated water. Rodent population control activities, including community rat-trapping drives, rodentproof food storage from recycled materials, and supervised garbage disposal systems, target one of the primary reservoirs of *Leptospira* bacteria.

Behavior changes and risk reduction strategies such as boot banks in areas with minimal flooding for shared protective gear, flood buddy systems for mutual safety checks, and local IEC patrols ensure that information and preventive measures reach residents during critical periods. Local surveillance and early detection enhance responsiveness through barangay (the smallest administrative division in the Philippines) case watch boards, regular health check days, and rapid reporting networks using SMS or social media to alert local health workers of suspected cases. Local production and distribution of protective and cleaning materials strengthen preparedness; soap-making workshops, DIY disinfectant production, and low-cost water filtration kits provide communities with essential tools even when external supplies are delayed.

Interventions embedded within a systems thinking framework become part of a feedback loop that connects grassroots actions to national policy. Community-driven measures address immediate, localized risks, whereas national-scale programs provide structural support such as infrastructure upgrades, health system readiness, and formal surveillance. That integration ensures that prevention is not solely dependent on costly, long-term government projects but is reinforced by sustained, everyday action at the community level, making leptospirosis control more adaptive, resilient, and sustainable over time.

## Conclusions

Leptospirosis prevention in the Philippines needs a shift from reactive responses to proactive, community-driven interventions. Integrating local, low-cost interventions within a systems thinking framework complements national programs and fosters sustainability. Some of those local interventions include community drainage clearing operations, rodent control, and grassroots surveillance. Linking community action with national and local policy coordination, research productions, and infrastructure investment creates reinforcing feedback loops that enhance resilience and reduce disease prevalence. Through this integrated approach, leptospirosis interventions become more adaptive, participatory, and effective in protecting vulnerable communities.

Appendix Additional information about rethinking leptospirosis prevention, the Philippines.
