# Supplementary material for: Rethinking Leptospirosis Prevention, the Philippines
Source: Emerg Infect Dis. 2026 Mar;32(3):397–403. doi: 10.3201/eid3203.251250 (PMC13016017; doi:10.3201/eid3203.251250)
Supplement: Appendix — Additional information about rethinking leptospirosis prevention, the Philippines. [file 25-1250-Techapp-s1.pdf]

Article DOI: https://doi.org/10.3201/eid3203.251250

EID cannot ensure accessibility for supplementary materials supplied by authors. Readers who have difficulty accessing supplementary content should contact the authors for assistance.

Rethinking Leptospirosis Prevention, the Philippines

Appendix

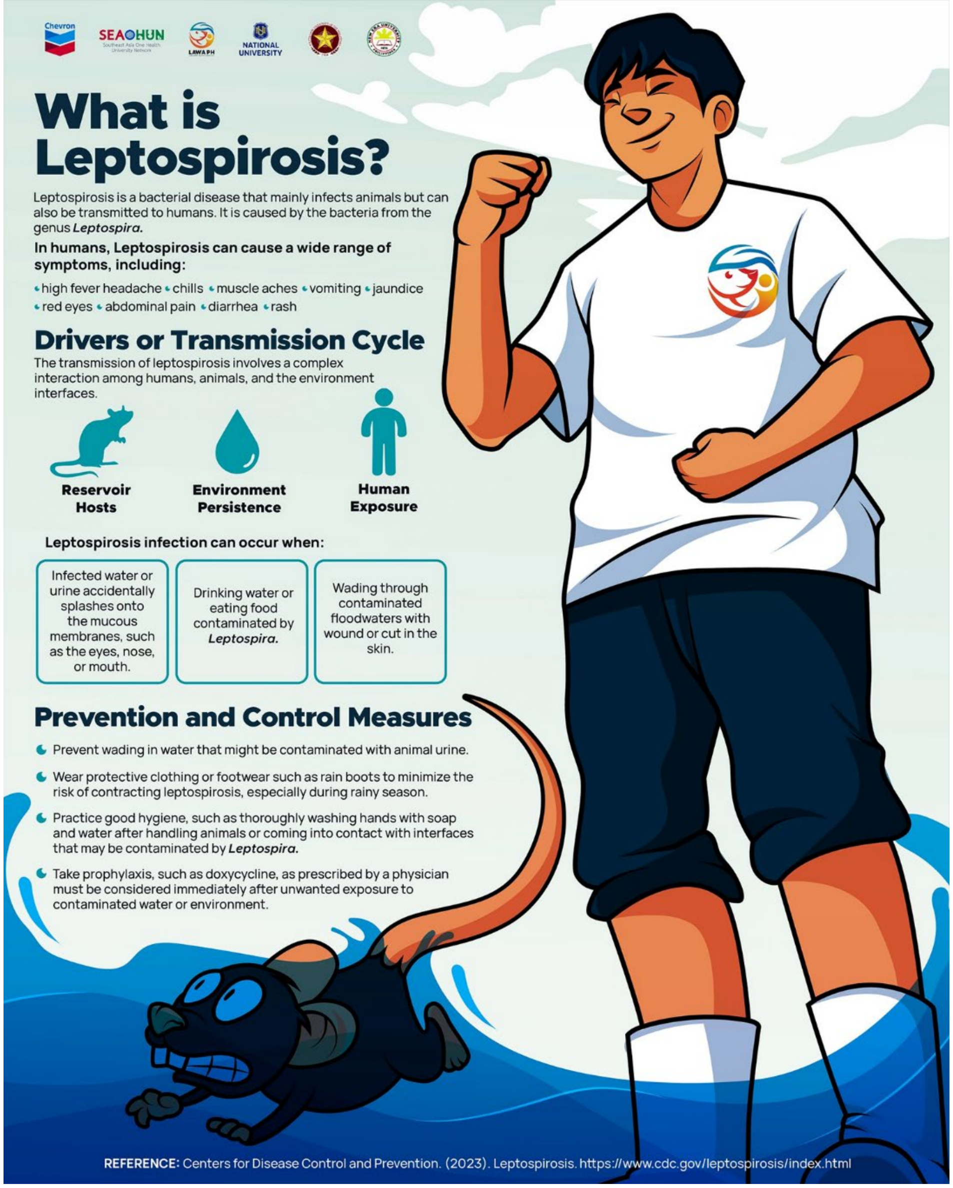

Appendix Figure. Information, education, and communication material created by Local Awareness, Watershed Action–Philippines and posted on its social media accounts.
